# Investigations of foreign bodies in the fore-stomach of cattle at Ngoma Slaughterhouse, Rwanda

**DOI:** 10.4102/jsava.v86i1.1233

**Published:** 2015-07-30

**Authors:** Borden Mushonga, Gervais Habarugira, Aline Musabyemungu, Jean C. Udahemuka, Festus I. Jaja, Dunisani Pepe

**Affiliations:** 1 Faculty of Agriculture and Natural Resources, University of Namibia, Namibia; 2 School of Animal Sciences and Veterinary Medicine, College of Agriculture, Animal Sciences and Veterinary Medicine, University of Rwanda, Rwanda; 3 Department of Livestock and Pasture Science, University of Fort Hare, South Africa

## Abstract

Ingestion of indigestible foreign bodies in cattle is a pathological condition of both economic and health importance. It is has mostly been reported in association with feed scarcity. The aim of this study was to investigate the occurrence and nature of indigestible foreign materials in abattoir fore-stomach specimens in Ngoma district, Rwanda. Each chamber was opened by incision, then given a thorough macroscopic examination by visual inspection and palpation for the presence of foreign materials. The results show that there is an overall occurrence of 17.4% foreign bodies in cattle. The highest occurrence (25.3%) was recorded in June (the driest month). Results further show that the majority of the foreign bodies were plastics (65.0%). More foreign bodies (29.5%) were found in older animals (5 years and above) than in younger and middle-aged animals (16.5 % and 6.0%, respectively). There was a higher prevalence of foreign bodies in female cattle (20.0%) than in males (15.7%). The presence of cassette tape, as observed in the study, has not been reported elsewhere. The high representation of plastics in animals (65.5%) in the light of a government plastic bag ban in supermarkets presents a major challenge to livestock production in Rwanda. What is disturbing is that it is not known if this problem is increasing or decreasing as there are no previous studies for comparison. However, the results will serve as a reference point for future studies to understand the true trend and true burden of plastic bags in livestock.

## Introduction

Ingestion of foreign bodies in cattle is a pathological condition of economic importance which leads to severe economic losses as a result of high morbidity and mortality rates (Radostits *et al*. 2007; Ramin *et al*. 2008). Because of their indiscriminate feeding habits, cattle are known to ingest and, at times, choke because of ingestion of different types of indigestible materials referred to as foreign bodies (Baumont 1996). The ingestion of indigestible materials has been associated with feed scarcity (Igbokwe, Kolo & Egwu 2003).

Depending on the nature of the ingested foreign bodies and the diagnostic facilities, the detection of foreign bodies in ruminants’ stomachs is routinely accomplished by exploratory surgery and, occasionally, by ultrasonography (Radostits *et al*. 2007; Ramin *et al*. 2008; Semieka 2010). However, a number of cases are diagnosed by radiology and at necropsy (Mozaffari, Olomi & Vosough 2009; Nugusu *et al*. 2013; Ramaswamy & Sharma 2011). Because cattle do not use their lips for prehension, they are more likely to ingest foreign bodies than small ruminants as they are more likely to eat chopped feed in which foreign bodies may be incorporated (Westwood 2011). Studies have shown that the non-penetrating foreign bodies commonly recovered in bovine stomachs are plastic bags, sack thread, ropes, leather, rubber, bed linen, pieces of lead pipe, straw baskets, hair and plant fibres (bezoars) (Anwar *et al*. 2013). The major penetrating foreign bodies include metallic pieces of wire, needles, nails and stones (Kahn & Line 2010; Nugusu *et al*. 2013; Ramaswamy & Sharma 2011). Most of these foreign bodies were found mainly in the fore-stomachs and they are responsible for most pathological conditions affecting this area (Tehrani *et al*. 2012). The presence of foreign bodies in the fore-stomachs of ruminants has gained attention in recent years and is a subject of attention worldwide, as it results in reduced production and, in some cases, death of animals (Igbokwe *et al*. 2003; Ramprabhu, Dhanapalan & Parathaban 2003; Remi-Adewunmi, Gyang & Osinowo 2004). In developed countries, industrialisation and agriculture mechanisation have further increased the occurrence of foreign bodies in ruminants, whilst in developing countries the high rate of occurrence is associated with poor farming management (Misk, Ahmed & Semieka 2004; Semieka 2010).

Notwithstanding the availability of information on foreign bodies in different animal species from elsewhere (Abdullahi, Usman & Mshelia 1984; Igbokwe *et al*. 2003; Remi-Adewunmi *et al*. 2004), such information has never been published in Rwanda. As recently as 2005, the Government of Rwanda, through the Rwanda Environmental Management Authorities (REMA), has banned the use of plastic bags for packaging in retail shops (Republic of Rwanda 2005). No studies have been carried out on prevalence of foreign bodies in cattle or the effects of the plastic ban on the environment and, consequently, on animal production and food security in Rwanda.

The objectives of this study were: to determine the occurrence of the foreign bodies in cattle; to describe the nature and patterns of occurrence of the various foreign bodies found in the stomachs of the cattle; to localise the foreign bodies in the various chambers of the stomach; to correlate the incidence of the foreign bodies with the body condition of animals; and to correlate the frequency of foreign bodies with animal factors such as age, breed and gender.

## Research method and design

### Setting

The study was conducted in Ngoma, one of the seven districts of the Eastern Province of Rwanda. The district is located at 2°09’30”S and 30°32’35”E. The average altitude ranges between 1400 m a.s.l. and 1700 m a.s.l. and an average annual rainfall of 1400 mm per year, which varies depending on the season (dry versus rainy season). The annual average temperature is around 20 °C.

### Materials

A total of 1261 cattle slaughtered at Ngoma slaughterhouse were used in this study. The cattle production system is extensive herding/grazing and night kraaling. At the slaughterhouse, an ante-mortem examination was performed with emphasis on assessment of body condition. It was evaluated based on a five-point scale ranging from ‘1’–‘5’, representing emaciated, poor, acceptable, fat and very fat or obese animals, respectively, as described by Roche *et al*. (2004) and Heinrichs and Ishler (1989).

### Design

A cross-sectional study was carried out at Ngoma slaughterhouse in the Eastern Province, Rwanda. The study was conducted over a period of 6 months from May to October 2012. The sample consisted of all cattle that came to the slaughterhouse during the period of the study.

### Procedure

After slaughter, the fore-stomach (comprising the rumen, reticulum and omasum) and the true stomach (abomasum) were carefully removed from the abdomen and placed in a container in such a way as to minimise spillage of contents from the different chambers. Each rumen, reticulum, omasum and abomasum was opened individually by incision and given a thorough macroscopic examination by visual inspection and palpation for the presence of foreign materials. All contents in each of the different chambers were examined thoroughly and foreign materials (bodies) in each chamber noted and recorded.

### Analyses

Descriptive statistics were used for analysis of both demographic and epidemiological characteristics. Categorical variables were described using percentages whilst bivariate analysis was performed using *χ*^2^ and Fischer's exact tests. Collected data were entered and managed in MS Excel and STATA 12.1 (Stanford University, CA 2012) and *p*-values ≤ 0.05were considered significant.

## Results

### Overall occurrence of foreign bodies

During the 6 months of the study, 17.4% (*n* = 1261) of the 1261 cattle slaughtered at Ngoma slaughterhouse and examined for probable presence of foreign bodies had foreign bodies in their stomachs. The types of foreign bodies were metallic objects such as nails, keys, and needles. The non-metallic objects were plastic bags, pieces of cloth, hairballs, stones, cassette-tape ribbons and cords from tires. The most commonly-observed foreign material was plastic, which occurred in 65.5% (*n* = 220) of those with foreign bodies in their stomachs.

The occurrence of foreign bodies was high in June and lowest in July (Table 1). The statistical analysis showed that there is a significant difference in the occurrence of foreign bodies according to months (*p* < 0.05,* n =* 1261).

**TABLE 1:** Occurrence of foreign bodies by month.

Period	Number of slaughtered animals	Animals with foreign bodies	Occurrence (%)
May	100	19	19.0
June	146	37	25.3
July	158	21	13.3
August	266	50	18.8
September	292	41	14.0
October	299	52	17.4
Total	1261	220	-

As indicated in Table 2, the highest occurrence of 29.4% (141/480) was found in animals aged 5 years and above. The lowest occurrence was observed in animals aged between three and 5 years, with an occurrence of 6.0%. The difference in occurrence of foreign bodies between age groups was statistically significant (*p <* 0.05,* n* = 1261).

**TABLE 2:** Occurrence of foreign bodies according to age group.

Age group (years)	Number of slaughtered animals	Positive animals	Occurrence (%)
1–3	302	50	16.6
3–5	479	29	6.0
5 and over	480	141	29.4
Total	1261	220	-

With regard to occurrence of foreign bodies in association with gender, 20.0% (101/505) of female cattle tested were found positive whilst the occurrence in males was 15.7% (119/756) (Table 3). Statistical analysis showed that the occurrence of foreign bodies is not significantly associated with gender (*p* < 0.05).

**TABLE 3:** Occurrence of foreign bodies according to gender.

Sex	Number of slaughtered animals	Positive case	Occurrence (%)
Female	505	101	20.0
Male	756	119	15.7
Total	1261	220	-

The occurrence of foreign bodies was inversely proportional to body condition (Table 4). The highest occurrence was observed in animals in the worst body condition (thin category) with 38.6% (112/290) of foreign bodies, whilst the fattest animals had the lowest occurrence, namely, 3% (11/365). Statistically, there was a difference (*p* < 0.05) between Body Condition Score (BCS) and the occurrence of foreign bodies.

**TABLE 4:** Occurrence of foreign bodies in relation to body condition score.

Body condition score	Number of slaughtered animals	Positive animals	Occurrence (%)
Thin	290	112	38.6
Normal	606	97	16.0
Fat	365	11	3.0
Total	1261	220	-

Table 5 shows that plastic bags were the most prevalent foreign body type (65.5%, *n* = 220), followed by hairballs (15.5%, *n* = 220). The least prevalent were keys (0.5%, *n* = 220). The difference between the number of foreign bodies and the positive cases was because of the fact that, in some animals, more than one type of foreign body was recovered.

**TABLE 5:** Occurrence of foreign bodies according to their nature (type).

Nature of the foreign bodies	Total	Percentage (%)
Plastic bags	144	65.5
Cloth	16	7.2
Nails	2	0.9
Key	1	0.4
Needles	2	0.9
Ropes (plastics)	14	6.3
Hairballs	34	15.4
Stones	7	3.1
Cassette-tape ribbons	3	1.3
Total of foreign bodies	223	-
Total of positive cases	220	-

As indicated in Table 6, 82.1% (*n* = 223) of foreign bodies were recovered from the rumen whilst nothing foreign was found in the omasum. The difference in the occurrence of foreign bodies in the compartments of the fore-stomach and the abomasum was significant (*p <* 0.05, *n =* 223). A highly significant occurrence of foreign bodies in the stomach chambers was observed.

**TABLE 6:** Occurrence of foreign bodies according to their nature and location.

Nature	Location				Total number of foreign bodies
	Rumen	Reticulum	Omasum	Abomasum	
Plastic bags	144	0	0	0	144
Pieces of cloth	16	0	0	0	16
Ropes	14	0	0	0	14
Nails	0	2	0	0	2
Needles	0	2	0	0	2
Keys	0	1	0	0	1
Stones	0	7	0	0	7
Hairballs (soft)	0	0	0	28	28
Hairballs (hard)	6	0	0	0	6
Cassette-tape ribbons	3	0	0	0	3
Total	183	12	0	28	223

The results in Table 7 show that thin animals had mostly plastics and ropes, with 67.7% (90/133) and 93.0% (13/14), respectively. With regard to these two type of foreign bodies, there is a significant difference between different BCS (*p <* 0.05).

**TABLE 7:** Prevalence of the foreign bodies according to their type and the body condition score.

Nature of the foreign bodies	Body condition			
	Thin	Normal	Fat	Total (Positive animals)
Plastic bags	90	42	7	133
Pieces of cloth	6	8	1	15
Ropes	13	0	1	14
Nails	0	2	0	2
Keys	0	1	0	1
Needles	0	2	0	2
Hairballs	0	34	0	34
Stones	0	5	2	7
Cassette-tape ribbons	0	3	0	3
Plastic + pieces of clothes	1	0	0	1
Plastic bags containing food	1	0	0	1
Plastic rope + plastic bag + hairballs	1	0	0	1
Total	112	97	11	220
Percentage (%)	50.9	44.0	5.0	100.0

## Ethical considerations

Ethical approval (by official confirmation notice) for this study protocol was obtained from the Institutional Review Board of the School of Animal Sciences and Veterinary Medicine, College of Agriculture and Veterinary Sciences, University of Rwanda. The stomach examination procedures were performed by a qualified veterinarian assisted by meat inspectors through routine ante- and post-mortem inspection aimed at ensuring personnel safety. Slaughterhouse owners were informed about the study purpose and procedures and provided written consent prior to study procedures being carried out on their animals.

## Trustworthiness

All the data used in this paper were collected by well-trained investigators. Examinations were done in accordance with animal welfare as well as occupational health and safety guidelines as defined by Rwanda Veterinary Services. All the foreign bodies were identified with certainty, indicating that all the results presented in this manuscript are trustworthy.

## Discussion

This study revealed an overall occurrence of 17.4% of foreign bodies, which consisted mainly of plastics, in the stomachs of cattle slaughtered at Ngoma slaughterhouse in the Eastern province. These results are similar to those of Ramaswamy and Sharma (2011), in a study based on exploratory ruminotomy carried out in Ethiopia.

Because cattle have a poor selective grazing adaptation (Westwood 2011), a prevalence rate of 17.4% in this study is not extraordinary and a lower prevalence of 6.1% reported by Fromsa and Mohammed (2011) in small ruminants in Ethiopia is not difficult to understand. Nearly similar prevalence rates of 8.9% and 11% were reported in sheep and goats by Hailat *et al*. (1997) and Hailat *et al*. (1998) from Jordan. However, higher prevalence rates reported previously in small ruminants have been explained by the fact that in desert countries, farmers live closely with their animals. As a result, the animals frequently eat household waste and graze very close to the homestead and are thus exposed to a higher risk of ingesting indigestible materials (Ghurashi *et al*. 2009; Hayder, Bakhiet & Mohammed 2006; Igbokwe *et al*. 2003; Tiruneh & Yesuwork 2010).

The differences in prevalence rates observed in female (20.0%) and male (15.7%) cattle have also been reported by Tiruneh and Yesuwork (2010), who concluded that the higher prevalence rates of foreign bodies in female cattle were a result of the effect of drought on the production stages of the females that occurred in the year of their study. They alluded to the fact that pica, which caused the animals to pick up strange objects as food, is normally more frequent in pregnant than in non-pregnant animals. In our study, the higher prevalence in female cattle may be explained by the fact that females generally have a longer lifespan than males, as livestock farmers normally do not sell females because they reproduce and increase the herd size. In addition, beef cows in Rwanda are also milked and therefore spend more time around the homestead – this generates higher exposure to domestic foreign bodies. The difference in prevalence rate in the present study was not statistically significant.

In this study, older animals and animals with poor body condition were found to be most affected with indigestible foreign bodies. This scenario has also been reported previously (Fromsa & Mohammed 2011; Hailat *et al*. 1997; Hailat *et al*. 1998; Tesfaye & Chanie 2012; Tiruneh & Yesuwork 2010; Vanitha *et al*. 2010). It is impossible in the current study to conclude whether the foreign bodies caused the loss of weight in animals or that animals in poor body condition were likely to have pica and hence pick up strange objects as a result. The finding of more foreign bodies in older animals may be a result of the gradual accumulation of indigestible materials ingested over a prolonged period of time. It is equally possible that animals are in poor body condition as a result of ingestion of indigestible plastics that interfere with food digestion and absorption.

The more frequent occurrence of rumen and reticulum impaction in emaciated and thin animals might be attributed to the interference of the foreign body with the absorption of volatile fatty acids, causing reduced weight gain. The results of this study are in agreement with those of Tesfaye and Chanie (2012) in Jimma, South-west Ethiopia and Fromsa and Mohammed (2011) in East Shoa, Ethiopia.

Semieka (2010) in Egypt used radiography and found out that needles and nails were frequent. Fromsa and Mohammed (2011) recovered plastic, leather, wire and hairballs in buffaloes and cattle. Hailat *et al*. (1997) also recovered such indigestible foreign bodies as plastic bags, pins, nails, hairballs, ropes and leather. In this study plastic, hairballs, ropes, stones, needles, keys, nails, plastic bags and wire were recovered from the stomachs of cattle. This is not surprising, as these objects are in common usage in Rwanda. However, in the present study, cassette ribbons, which have not been reported previously in literature, were also observed.

The recovery of plastics as the most prevalent foreign body is as worrying as it is puzzling. Recently, the Government of Rwanda – through the REMA – banned the use of plastic bags in retail shops (Republic of Rwanda 2005). Rwanda is a country with a high population density, where man and animals are forced to share the limited space; animals graze where man carries out his activities of daily living. A scene of livestock grazing along roads and alleys full of plastics is not uncommon along major roads such as the Ngoma–Kigali or Kigali–Nyagatare roads in Rwanda. In fact it is not uncommon to witness a goat, sheep or cow actually chewing a plastic sheet. According to the Holland and Kezar (1995), foreign bodies may usually be found in the zones of strong human concentration (cities) in the cattle-breeding areas, around schools, dwellings, along the roads and in the market, where one finds the sachets, food bags, the pieces of fabric and scrap materials. They further state that the circumstances of ingestion of the foreign bodies can result from carelessness, when people dispose of indigestible materials on animal pasture or in the vicinity of the animals. Occasionally, when the animal body is deprived of certain nutrients, animals have a tendency to eat anything within their reach (pica).

Because of the issues discussed, the high incidence of plastics in the fore-stomach is hardly surprising. The fact that this scenario comes on the heels of the government-gazetted plastic paper bag ban may mean that the ban is not showing any positive results. However, the non-biodegradability of plastics may mean that the same plastics that were already present in the older animals are still there. Although it cannot be concluded that the present study has shed any light on the impact of the plastics ban by government, it is important to note that no such information has ever been published in Rwanda. It therefore warrants a wider follow-up assessment study of the effects of the plastic paper ban on the environment in Rwanda. If such follow-up studies could be carried out, then these results can serve as baseline information for comparison with their results.

The recovery of most foreign bodies in the rumen is also not surprising, as it has been reported previously in small ruminants (Fromsa & Mohammed 2011; Hailat *et al*. 1997; Igbokwe *et al*. 2003; Remi-Adewunmi *et al*. 2004; Tiruneh & Yesuwork 2010) and in buffaloes (Khan, Habib & Siddiqui 1999). This may be because almost all ingested feed goes to the rumen and most indigestible materials do not progress to other stomach chambers.

Furthermore, the sheep and goats slaughtered at Addis Ababa Abattoir cohabit with the traders for days and weeks before getting sold and may be exposed to grazing on garbage contaminated with plastic bags. Remi-Adewunmi *et al*. (2004) had reported a much higher prevalence rate (97%) in sheep and goats brought from urban areas of Nigeria for slaughter. Hayder *et al*. (2006) had also retrospectively studied the occurrence of foreign bodies in goats from Khartoum state and reported a prevalence of 52.9% in 2000 and 33.3% in 2001.

## Conclusion

The findings of this study indicated that littering the environment with plastic bags and other indigestible materials could pose health problems for free-ranging cattle unless people reduce disposal of plastics on pastures and begin to practise routine picking up and disposal of plastics from animal pastures. This study showed that there is association between age and prevalence of foreign bodies. More foreign bodies were found in the rumen (82%) than in the other compartments. This study revealed an overall prevalence of 17.4% of foreign bodies in the stomach.

One can conclude that there is a high prevalence of foreign bodies in cattle from the largely rural districts of Ngoma. One can only extrapolate that cattle in larger urban centres, such as Kigali and Musanze, where there are higher population densities, could have a much higher occurrence of foreign bodies. It has been highlighted by previous researchers that the presence of large numbers of foreign bodies in animals interferes with digestion and affects animal productivity. It is therefore reasonable to conclude that food security in Ngoma and, perhaps, in Rwanda, is threatened by plastic pollution. Whether the problem of foreign bodies in cattle in Rwanda is increasing or decreasing, only time and follow-up studies will tell.
